# Grand Challenges in Digital Health

**DOI:** 10.3389/fpubh.2015.00134

**Published:** 2015-05-05

**Authors:** Patty Kostkova

**Affiliations:** ^1^University College London (UCL), London, UK

**Keywords:** digital health, eHealth, digital revolution, mHealth, challenges, health games, Medtech

Today, everything is affected by the digital revolution – the impact of new technology on improving the health and well-being of individuals, communities, and populations is unprecedented. Recent technological achievements have revolutionized clinical practice, from prevention through diagnosis, monitoring to disease management, and enabled unprecedented public interest and engagement in self-management and well-being. *Digital health* is defined as the “use of information and communications technologies to improve human health, healthcare services, and wellness for individuals and across populations.”

Dozens of digital health projects have been conducted in Europe alone (1) and with the growth of mobile technology for improving health and well-being (mHealth). There is an unprecedented opportunity to transform the healthcare sector and empower citizens in taking charge of their own health (2).

However, the successful development, integration and implementation of new technology and methods require a radical shift from traditional and single-disciplinary academic and clinical approaches. In order to truly embrace these opportunities and transform healthcare and improve well-being, we need a new approach to science and health research. Only when investigated together, aimed at solving real-world problems, will health and technology be in a position to create results with significant impact on the delivery of clinical and social care and improve the well-being of individuals and populations.

First, recent technological advances enabled by the creation of real-time big data streams, social media, and infectious disease modeling are the focus of public health computer science, aiming to strengthen disease surveillance, early-warning, preparedness, and response through integration of traditional surveillance systems with new big data sources. Second, advances in reliability and accuracy of medical devices and personalized technology, together with booming wearable and tracking technology (MedTech), have rapidly become established as the mainstream enhancing opportunities for personalized care, improving self-management, and bringing the desirable outcome: behavior change. Third, with over 4.55 billion people worldwide using a mobile phone in 2014, mHealth apps and interventions empower users in the developed world and are accelerating unprecedented access to best evidence and healthcare service in the low and middle income settings. Fourth, more than a decade ago, Sir Muir Gray forecasted that “*knowledge is the best enemy of disease – the application of what we know already will have a bigger impact on health and disease than any drug or technology likely to be introduced in the next decade*” (3). Better use of technology for capturing, understanding, and disseminating knowledge is paramount for fulfilling this vision. The recent research achievements in web science, data mining and analytics, medical ontologies, and recommender systems provide further opportunities for better evidence dissemination, medical advice, and development of personalized persuasive intelligent systems. Fifth, serious games for health- and game-based learning have crossed the rubicon from entertainment technology to education and health interventions, and are getting firmly established as health educational and intervention tools. Finally, in light of the speed of data sharing technologies and the absence of a legal framework, we must engage in policy debates safeguarding individual privacy, regulation usage of data for commercial purposes, while enabling transparent data sharing for research.

However, these challenges are only achievable through truly multi-disciplinary research collaborations.

## Multidisciplinary Digital Health

Intrinsically a multidisciplinary domain, digital health spans disciplines including computer science, engineering, information science, journalism, economy, clinical medicine, public health, epidemiology, and others. Technology and computer science investigate new frameworks and algorithms of excellent theoretical value, however, for successful digital health research. It is essential to address real-world medical challenges, solve clinical or public health problems, and recognize patients’ needs. Similarly, clinical IT solutions should improve management of conditions and delivery of care through utilization of cutting edge computer science solutions. This gap needs to be bridged by a joint multidisciplinary collaboration between healthcare professionals/medical scientists and computer scientists/engineers; so, both professions contribute to join research at equal levels. Thus, this Digital Health section, linked from *Frontiers in Public Health* and *Frontiers in ICT*, champions this approach by providing a unique interdisciplinary publication venue for both professional communities, enabling the cross-fertilization and breeding of the next generation of versatile researchers who truly appreciate the language, methods, needs, and challenges of other disciplines. This momentum is creating the need for interdisciplinary health and public health computer science training. Closer partnerships between research and industry/start-ups bringing on board policy makers are essential to drive a real change.

## Big Data and Public Health

Recent achievements in mobile technology and sensor/wearable devices have created real-time geo-located big data streams, facilitating context-aware social media communication and participatory systems that are radically changing the way we monitor populations with unprecedented opportunities for disease surveillance, early-warning, preparedness, and rapid response. Public health surveillance decision making is being revolutionized by the mining, analysis, and visualization of heterogeneous data sources, but must better leverage cutting-edge computer science to integrate with existing national and international surveillance systems (4). The potential of big data for monitoring population mobility using mobile phones to fight human-transmitted infections has also proven promising, such as in 2009 H1N1 outbreak in Mexico and Ebola in 2014 (5). However, integrating experimental surveillance systems [e.g., Influenzanet (6), MediBoard (7, 8)], with established news monitoring engines [MediSyS (9), GPHIN (10), HealthMap (11)], and national and international surveillance systems (including animal surveillance systems with the vision for a “One Health” approach), remains a major challenge. Technology support for threats validation and verification, risk assessment, and evaluation could significantly reduce human efforts currently provided by public health experts. Moreover, risk communication during emergencies and about health threats increasingly takes place online and on social media, rather than through controlled official traditional health communication paradigms. Effective use of these new channels by public health agency for citizens engagement and risk communication is still lagging behind technological progress (12).

## MedTech, Self-Management, and Personalized Care

Enabled by the recent deployment of innovative medical devices, healthcare provision is being increasingly driven outside clinical settings to community and home and even further to care provision on-the-go (13). While integration of devices to hospital care in dynamic IT “ecosystems” replacing traditional large enterprise databases is lagging behind due to complex regulatory frameworks, major industries have increased their presence in the digital health domain aimed at personal/non-clinical care. In addition to the traditional pharmaceutical and MedTech industries, for example, Coca-Cola and Verizon are adopting health initiatives (14), IT (Google, Apple) and telecommunications are competing for fast growing wearable/tracking devises markets (Apple ResearchKit, launched in March 2015 presents a new attempt in competing for the developer tool making it easier for medical researchers to use apps to collect data for clinical research, raising the importance of data privacy concerns even higher).

By 2018, it is predicted that 75 million wireless-connected health and fitness devices will be shipped, up from 23 million in 2011 (15). Mobile technology with self-monitoring/tracking and wearable devices interacting directly with social media, has dramatically increased citizens’ engagement with healthy lifestyles and well-being, and changed the way patients are empowered to independently monitor and self-manage their conditions [for example, players such as MC10, HealBe, and Proteus are transforming the use of sensors and wearables to improve health outcomes (16)]. Nevertheless, more research is required to fully understand the impact and design regulation for this new technology, which is supported by scientific robust evidence.

Finally, novel approaches to human–computer interaction (17) and better understanding of human error in the “real” clinical context (18), as well as long-term sustained user engagement, are required to improve personal motivations, commitment to self-management, and treatment adherence to bring the desirable aims – improve healthcare outcomes and promote behavior change (19).

## mHealth and Global Health Interventions

It is estimated there are around 4.55 billion people worldwide using a mobile phone in 2014, of which about 1.75 billion are smartphone users (20). The estimated forecast of the mhealth market was $6.7 billion at the end of 2014 (21). The use of mobile phones for accessing information about health has almost doubled since 2010 (when it was 17%) to 31% in 2013. According to Pew Research (22), this includes 52% of smartphone owners. Inevitably, the number of health apps is growing every day – 93% of doctors believe that apps can improve health outcomes and the same number also see benefits from connecting health apps to patients’ electronic personal records (EPRs), according to a study conducted by eClinicalWorks (23). mHealth can enable policymakers and healthcare practitioners to contact a large number of people with a high degree of accuracy, ensuring information is provided to those who need it, when it is needed (2). While the potential of mHealth is enormous, integration into the IT clinical infrastructures with the successful resolution of privacy and security issues remains an ongoing challenge. Regulatory frameworks and evidence for the actual impact on clinical care and quantifiable improvement of health outcomes as a result of mHealth are also limited.

While mobile adoption is generally slowing, new users in the developing regions of Asia-Pacific and the Middle East and Africa will drive further increases (20) accelerating user empowerment and enabling unprecedented access to best evidence and healthcare services in the developing world (24). Recently, mHealth innovations and a variety of applications have been enhancing the delivery of care and a number of solutions have demonstrated the potential of technology to bring better health to the poorest parts of the world (25). However, efforts in the developing world are subject to additional challenges including sustainable implementations; the difficulties in achieving interoperability and other barriers have been identified as obstacles to sustainable uptake (26). There is a need for more robust studies producing evidence on tangible quantifiable health outcomes and broader impact on citizens’ health and well-being (27).

## Evidence and Knowledge: Semantics, Social Media, and Persuasion

*“Knowledge is the enemy of disease, the application of what we know will have a bigger impact than any drug or technology likely to be introduced in the next decade”* famously predicted Sir Muir Gray, Director of the UK NHS National Knowledge Service and NHS Chief Knowledge Officer over 10 years ago (28), when he established the National Electronic Library for Health in the UK (29). However, despite the recognition of the importance of access to the latest evidence and recent research achievements in computer science knowledge management, wide adoption and standardization for clinical care systems remain a challenge. Research into Web science, medical ontologies, and taxonomies (30, 31), and their authoring (32) enables better access to evidence through semantic search and navigation (33). The application of recommender systems to health resources provides further opportunities for better evidence dissemination, instant access to medical advice, and development of personalized intelligent systems for patients and healthcare professionals. However, these technologies need to be underpinned by better understanding and profiling of user needs in order to customize information access (34) and demonstrate the desirable impact on knowledge and attitude change (35). Although the use of social media for discussing important health issues such as antibiotics (36) has recently become mainstream behavior for citizens (37, 38), it provides a novel opportunity for real-time risk communication for health authorities especially during public emergencies (12). Nevertheless, robust social media use supported by evidence-based guidelines is still in its infancy. Agent-based argumentative persuasive technology provides an avenue for improved engagement and potential behavior change (39), but more research is needed to demonstrate the impact of these achievements on clinical results.

## Serious Health Games and Games-Based Learning and Training

The emerging field of serious health games is changing the landscape of health education and user engagement with game-based learning (GBL), based on problem-based learning (PBL) approaches that have been well established in medical education for decades. GBL has been enjoying unprecedented interest among researchers (40) impacting those with chronic conditions (41), as well as improving awareness of hygiene practices and well-being (42). However, little interest has been given to investigating methods for the assessment of their effectiveness, using both computing and clinical methodologies, to demonstrate tangible impact on personal health, knowledge, attitude and behavior change, and ultimately health outcomes. Game evaluation focus remains on investigating usability and technical aspects. Further research is required to move serious games from pilot feasibility projects to recognized evidence-based health interventions and improve game-based learning authoring tools to make them accessible for not-expert authors (such as the eAdventure platform) (43). Online medical training must also further explore the role and engagement of communities of practice (CoP) (44) rather than focus only on providing knowledge to fully embrace the potential of the technology and e-learning.

## Personal and Population Data – To Share or Not to Share?

Research on EPR, real time big data streams generated by social media, and increasingly popular tracking and wearable devices has demonstrated the potential to improve health outcomes and provide signals for early warning. Developments such as crowdsourcing, participatory surveillance, patients pledging to become “data donors,” and the ‘quantify self’ movement (where citizens share data through mobile device-connected technologies) have been significant game-changers in terms of data sharing. However, in the absence of transparent regulations and governance structures, and increasing exploiting of data collected via apps, searchers by industry, there are two strikingly disparate approaches to data ownership, responsibility over sharing and accountability.

On one hand, traditionally risk adverse governments seem failing to communicate the advantages and opportunities arising from sharing anonymized patients data for research purposes to their citizens. In the UK, the failure in communication and lack of transparency over the control of data ownership resulted in loss of citizens’ trust and the backlash over the proposed implementation of care data initiative enabling large NHS patient data sharing (45, 46).

On the other hand, citizens seem less concerned about their more accurate and potentially private health data being directly collected by major IT companies and MedTech manufactures through tracking/wearable devices and social media for data-driven marketing purposes. Recent study by Libert investigating top 50 search results for 2000 common diseases identified that a full 91% of servers made the user search available to outside companies – that included not only commercial sites but government organizations, universities, and non-profits too (47). As medical data are of amorous commercial value, cyberattacks on medical sites holding patients data are on rise (48).

Third, population-level epidemiological datasets are also collected through surveillance systems. While notification of potential threats is enforced at international level through the International Health Regulations, IHR) and at European level with ECDC (EC Decision 2008/426/EC), countries remain in control of the datasets while surveillance population level data would be invaluable for scientific research, epidemic intelligence, and early-warning and risk assessment. Legal frameworks and operational procedures limiting the sharing of surveillance and epidemiological data between agencies and systems, as well as research organizations, are becoming openly challenged by open data initiatives in the public health domain. These could potentially enable almost real-time data sharing and faster response during emergencies, while opening new frontiers for data-driven interdisciplinary research.

As the key challenge – transparent access to health data – remains pushed aside, scientific and healthcare communities must engage with the underlying political, human, and legal challenges to enable and facilitate transparent data access for research needs and large-scale integrations. As personal data are subject to industry-defined terms of conditions, much needed oversight and international government regulations are required to restore user control of personal data with implications for public policy supporting a balanced agenda that safeguards personal information, while enabling the use of data to improve health.

## Conclusion

The “Digital Health” section of *Frontiers in Public Health* will provide the ideal publication venue for world-class interdisciplinary research addressing these, and many more challenges arising in the future with curiosity, and the desire to transform healthcare and impact the health of citizens and populations globally. Through the cross-fertilization of ideas and growth of an involved and engaged multi-disciplinary research community, the innovative research published in the “Digital Health” section will contribute to transforming how care and public health are delivered and accessed over the next decade.

## Conflict of Interest Statement

The author declares that the research was conducted in the absence of any commercial or financial relationships that could be construed as a potential conflict of interest.
